# Associaton of Retinol Binding Protein 4 (RBP4) Levels With Hyperuricemia: A Cross-Sectional Study in a Chinese Population

**DOI:** 10.3389/fendo.2022.879755

**Published:** 2022-06-29

**Authors:** Guo-bao Hong, Xiao-fei Shao, Jia-min Li, Qin Zhou, Xiao-Su Ke, Pei-Chun Gao, Xiao-Lin Li, Jing Ning, Hai-Shan Chen, Hua Xiao, Chong-Xiang Xiong, Hequn Zou

**Affiliations:** ^1^ Department of Nephrology, The Affiliated Shunde Hospital of Jinan University, Foshan, China; ^2^ Department of Nephrology, Nanhai Distric People's Hospital of Foshan, Foshan, China; ^3^ Department of Nephrology, Institute of Nephrology and Urology, The Third Affiliated Hospital of Southern Medical University, Guangzhou, China; ^4^ Department of Nephrology, Guangdong Electric Power Hospital, Guangzhou, China; ^5^ Nanjing CR Medicon Pharmaceutical Technology Co., Ltd, Nanjing, China; ^6^ Department of Nephrology, South China Hospital of Shenzhen University, Shenzhen, China; ^7^ Department of Nephrology, The First Affiliated of Dongguan, Guangdong Medical University, Dongguan, China

**Keywords:** retinol binding protein, hyperuricemia, prediction, cross-sectional survey, risk

## Abstract

**Background:**

There are few studies on predictive biomarkers for hyperuricemia, and the predictive value of these biomarkers tends to be poor. Additionally, no reports have described the predictive value of retinol binding protein 4 (RBP4) for hyperuricemia.

**Purpose:**

This study was performed to evaluate the value of RBP4 for predicting the risk of hyperuricemia in a general population, determine whether RBP4 could be used alone or in combination with other factors to predict the risk of hyperuricemia in the general population, and establish an optimum predictive model.

**Methods:**

We conducted a population-based cross-sectional survey in 2018, involving a questionnaire, physical examination, and laboratory testing. We enrolled 2303 individuals by stratified random sampling, and 2075 were included in the data analysis after applying the eligibility criteria.

**Results:**

Serum RBP4 level had a highly significant association with hyperuricemia (*P*<0.001). After adjusting for potential confounders, logistic regression indicated that the risk of hyperuricemia was highest in the highest RBP4 quartile (odds ratio: 7.9, 95% confidence interval [CI]: 4.18–14.84, compared to the lowest quartile). The area under the receiver operating characteristic (ROC) curve (AUC) for RBP4 was 0.749 (95% CI: 0.725–0.774, *P*<0.001), which was higher than that for all the other predictors assessed. The optimum model for predicting hyperuricemia in the general population consisted of RBP4, sex (male), body mass index, serum creatinine, high-sensitivity C-reactive protein, fasting blood glucose, insulin, and alcohol consumption. The AUC was 0.804 (95% CI: 0.782–0.826, *P*<0.001).

**Conclusions:**

RBP4 is strongly associated with hyperuricemia, and its predictive value was higher than that of traditional predictors.

## Introduction

The global prevalence of hyperuricemia has increased rapidly in the past few decades (1–3); it is 14.6–20% in the US (4) and 13.3–18.4% in China, and it is more common (25.5%) in southern China (5–8). Our 2012 epidemiological survey in Zhuhai city showed that the prevalence of hyperuricemia reached a staggering 32.4%, which is the highest for any location assessed in China (9). In addition to causing gout, numerous studies have shown that hyperuricemia increases the risk of metabolic syndrome (MetS) (10, 11), chronic kidney disease (12), acute kidney injury (13), hypertension (14), cardiovascular disease (15), and cerebral infarction (16).

Unfortunately, studies on the risk and predictors of hyperuricemia are rare. Although some groups have reported on factors (age, body mass index [BMI], waist circumference [WC], triglycerides [TG], etc.) (7, 17) that influence hyperuricemia, few studies have focused on individual biomarkers (visceral adiposity index (18), TG-glucose index (19), etc.) or models for predicting hyperuricemia (20–22). Additionally, most reported predictors have poor performance or are not suitable for large-scale clinical use because of issues such as cost and complexity.

Retinol binding protein-4 (RBP4) was recently recognized as a type of adipokine (23). Several small-sample studies have reported an association between RBP4 and hyperuricemia in patients with diabetes, chronic kidney disease, and MetS, but none of them focused on the general adult population (24, 25). To our knowledge, there are no studies on the predictive value of RBP4 for hyperuricemia.

This large-scale study aimed to explore the association between RBP4 and hyperuricemia in the general population, and to evaluate whether RBP4 can predict hyperuricemia. We also hope that in the absence of serum uric acid results, abnormally elevated levels of RBP4 may suggest the importance of further serum uric acid testing.

## Methods

### Study Population

A cross-sectional survey of the general population in Wanzai town in Zhuhai city, China, was conducted in 2018. Using stratified random sampling, 2303 adults who were permanent residents of Wanzai town were initially enrolled in the study [1054 in the first visit in 2018 and another 1249 later in 2018 (the second visit was the follow-up visit of a similar epidemiological survey that we conducted in 2012 in Wanzai town)]. We followed the same sampling procedures described in previous reports on our 2012 epidemiological survey (26–30).

The exclusion criteria were as follows: (1) treatment with uric acid-lowering drugs or drugs that affect uric acid in the last 6 months, (2) severe liver or kidney damage, (3) acute cardiovascular or cerebrovascular diseases, and (4) pregnancy or breastfeeding.

After applying the eligibility criteria, 2075 individuals were included in the final analysis.

### Ethics Approval

The study was approved by the Ethics Committee of the Third Affiliated Hospital of Southern Medical University and was conducted in accordance with the Declaration of Helsinki. All subjects provided written informed consent at recruitment.

### Data Collection

Data on sociodemographic factors (age, sex, and education level), lifestyle (physical inactivity [physical activity <1 time/week], current smoking, and current alcohol consumption [≥1 time/week]), medical history (hypertension, diabetes, coronary heart disease [CHD], and stroke), and medication use were collected using a questionnaire. The detailed design of this study (including the urine specimen collection) was the same as in our previous epidemiological survey conducted in 2012 (26–30).

Physical examinations were performed to collect data on blood pressure (systolic blood pressure [SBP] and diastolic blood pressure [DBP]), weight and height (which were used to calculate BMI), and WC. BMI was used to define obesity according to Chinese obesity criteria. Normal weight was defined as BMI<24 kg/m^2^. Obesity was defined as BMI >=28 kg/m^2^, BMI 24-28 kg/m^2^ was overweight.

Blood specimens were collected after overnight fasting, stored at 2–8°C immediately after collection, and transported to the Central Laboratory of the Third Affiliated Hospital of Southern Medical University within 3 hours (31). RBP4 levels were assessed using an immunoturbidimetric method (Shanghai Beijia Biochemical Reagent Company, Shanghai,China). Hyperuricemia was defined as ≥420 μmol/L (7 mg/dL) in males and ≥360 µmol/L (6 mg/dL) in females (32). The following parameters were also measured(apparatus: Roche Cobas c501, Penzberg, Germany): TG, high-density lipoprotein (HDL), low-density lipoprotein (LDL), fasting blood glucose (FBG), insulin, homeostasis model assessment of insulin resistance (HOMA-IR, defined as: (FBG×insulin)/22.5), serum creatinine (SCr), cystatin C, high-sensitivity C-reactive protein (hsCRP), interleukin (IL)-6, serum uric acid, and estimated glomerular filtration rate (eGFR, defined according to CKD-EPIscr formula). Urinary albumin-to-creatinine ratio (ACR), N-acetyl-β-D-glucosaminidase (NAG), and β2 microglobulin (β2MG) were also assessed.

### Statistical Analysis

Continuous variables with a normal distribution are presented as the mean and standard deviation, while those with a non-normal distribution are presented as the median and interquartile range. Categorical variables are presented as frequencies and percentages. Continuous variables were compared between groups using independent-samples t tests or analyses of variance (for normally distributed variables) or Mann-Whitney U tests (for non-normally distributed variables). Categorical variables were compared between groups using χ^2^ or Fisher’s exact tests.

Six binary logistic regression models (stepwise conditional), with hyperuricemia as the independent variable and RBP4 quartile as the independent variable, were used to calculate odds ratios (ORs) and 95% confidence intervals (CIs). The regression models adjusted for the following covariates: (1) age and sex; (2) model 1 covariates plus hypertension, diabetes, CHD, education of high school or above, physical inactivity, current smoking, and current alcohol consumption; (3) model 2 covariates plus SBP, DBP, log TG, LDL, HDL, BMI, eGFR, FBG, and log insulin; (4) model 2 covariates plus SBP, DBP, LDL, HDL, BMI, eGFR, and log HOMA-IR; (5) model 4 covariates plus log hs-CRP, and log IL-6; and (6) model 5 covariates plus log NAG and log ACR. SCr is strongly correlated with eGFR, so it was not included in the regression models.

The area under the receiver operating characteristic (ROC) curve (AUC) was used to assess the predictive value of RBP4, other predictors, and the following three predictive models for hyperuricemia: (1) RBP4, sex, BMI, SCr, log hs-CRP, log insulin, log HOMA-IR, and current alcohol consumption; (2) RBP4, sex, BMI, SCr, log hs-CRP, and FBG; and (3) RBP4, sex, BMI, SCr, log hs-CRP, FBG, log insulin, and current alcohol consumption. Youden’s index (sensitivity + specificity – 1) was used to select the optimum cutoff value of RBP4. The reciprocals of HDL and eGFR were used due to their negative associations with serum uric acid level.

Statistical analyses were performed in SPSS software version 20.0 (IBM Corp., Armonk, NY, USA). A two-sided *P* value <0.05 was considered statistically significant.

## Results

The baseline characteristics of the participants are shown in **Table 1**. Those in the hyperuricemia group were more likely to be male and older compared to those in the non-hyperuricemia group, and they had higher rates of hypertension, diabetes, CHD, alcohol consumption, and physical inactivity (*P*<0.05). In addition, there were higher values of SBP, DBP, WC, BMI, TG, LDL, FBG, insulin, HOMA-IR, SCr, cystatin C, NAG, hs-CRP, IL-6, and RBP4 in the hyperuricemia group compared to the non-hyperuricemia group (*P*<0.05), but lower values of HDL, eGFR, and serum uric acid (*P*<0.001).

**Table 1:** Participant baseline characteristics.

Characteristic	Total	Hyperuricemia	Non-hyperuricemia	*P*
	*n*= 2075	*n*= 651	*n*= 1424	
Sex, male (%)	744 (35.9)	297 (45.6)	447 (31.4)	<0.001
Age (years)	55.7±13.4	58.2±13.4	54.6±13.3	<0.001
History of hypertension (%)	615 (29.6)	254 (39.0)	361 (25.4)	<0.001
History of diabetes (%)	157 (7.6)	62 (9.5)	92 (6.7)	0.02
History of CHD (%)	77 (3.7)	35 (5.4)	42 (2.9)	0.01
History of stroke (%)	30 (1.4)	18 (1.8)	12 (1.3)	0.31
Education of high school or above (%)	702 (36.9)	202 (33.9)	500 (38.3)	0.07
Physical inactivity (%)	759 (36.6)	213 (32.7)	546 (38.3)	0.01
Current smoking (%)	249 (12.5)	91 (14.5)	158 (11.6)	0.06
Current alcohol consumption (%)	96 (4.9)	44 (7.1)	52 (3.9)	0.002
Weight status				
normal weight	1040 (51.0)	221 (34.6)	819 (58.4)	<0.001
overweight	724 (35.5)	280 (43.8)	444 (31.7)	
obesity	277 (13.6)	138 (21.6)	139 (9.9)	
SBP (mmHg)	134.2±19.8	139.4±19.1	131.7±19.7	<0.001
DBP (mmHg)	82.5±10.7	85.4±10.7	81.2±10.4	<0.001
WC (cm)	84.7±9.9	89.0±9.2	82.7±9.6	<0.001
BMI (kg/m^2^)	24.2±3.5	25.6±3.5	23.6±3.3	<0.001
TG (mmol/L)	1.29 (0.96–1.85)	1.67 (1.22–2.39)	1.18 (0.89–1.62)	<0.001
LDL (mmol/L)	3.22±0.94	3.31±1.00	3.19±0.91	0.01
HDL (mmol/L)	1.51±0.35	1.40±0.33	1.56±0.34	<0.001
eGFR (mL/min/1.73 m^2^)	84.9±16.8	77.4±17.9	88.3±15.0	<0.001
hs-CRP (mg/L)	1.38 (0.55–2.55)	1.86 (1.04–3.43)	1.18 (0.19–2.15)	<0.001
FBG (mmol/L)	5.26±1.22	5.37±1.07	5.21±1.28	<0.001
HOMA-IR (µU/mL ·mmol/mL)	2.06 (1.42–3.15)	2.61 (1.82–3.87)	1.83 (1.30–2.79)	<0.001
Insulin (μU/mL)	9.1 (6.6–13.1)	11.5 (8.1–15.7)	8.3 (6.1–11.8)	<0.001
Serum creatinine (µmol/mL)	77.1±19.5	85.8±24.0	73.1±15.6	<0.001
RBP4 (mg/L)	56.2±14.9	66.2±16.1	51.7±11.8	<0.001
Serum uric acid (µmol/L)	349.7±90.1	450.0±65.4	303.9±56.3	<0.001
ACR (mg/g)	10.7 (6.9–19.0)	8.0 (5.7–12.0)	10.6 (7.0–19.6)	0.54
Cystatin C (mg/L)	0.93±0.23	1.03±0.28	0.88±0.18	<0.001
NAG (U/L)	2.8 (1.50–5.00)	3.00 (1.70–5.50)	2.70 (1.50–4.70)	0.01
IL-6 (pg/mL)	3.23 (2.57–4.37)	3.48 (2.78–4.62)	3.11 (1.50–4.20)	<0.001
β2MG (µg/mL)	0.09 (0.05–0.16)	0.10 (0.06–0.19)	0.08 (0.05–0.14)	0.77

Data are shown as mean±standard deviation, median (interquartile range), or frequency (percentage).

ACR, urinary albumin-to-creatinine ratio; β2MG, β2 microglobulin; BMI, body mass index; CHD, coronary heart disease; DBP, diastolic blood pressure; eGFR, estimated glomerular filtration rate; FBG, fasting blood glucose; HDL, high-density lipoprotein; HOMA-IR, homeostasis model assessment of insulin resistance; hs-CRP, high-sensitivity C-reactive protein; IL-6, interleukin 6; LDL, low-density lipoprotein; NAG, N-acetyl-β-D-glucosaminidase; RBP4, retinol binding protein 4; SBP, systolic blood pressure; TG, triglycerides; WC, waist circumference.

The participants were divided into quartiles based on RBP4. Those in the fourth quartile were more likely to be male and older compared to the participants in the first quartile, and they were less educated and had higher rates of hypertension, diabetes, smoking, alcohol consumption, and physical inactivity (*P*<0.05). The values of SBP, DBP, WC, BMI, TG, LDL, FBG, insulin, HOMA-IR, serum uric acid, SCr, cystatin C, NAG, hs-CRP, IL-6, and ACR were higher and the values of HDL and eGFR were lower in the fourth quartile compared to the first quartile (**Table 2**).

**Table 2:** Participant baseline characteristics by RBP4 quartile.

Characteristic	Quartile 1	Quartile 2	Quartile 3	Quartile 4	*P*
	(≤45.9 mg/L)	(46.0–54.0 mg/L)	(54.0–64.6 mg/L)	(>64.6 mg/L)	
	*n*= 518	*n*= 519	*n*= 521	*n*= 517	
Sex, male (%)	106 (20.5)	172 (33.1)	218 (41.8)	248 (48.0)	<0.001
Age (years)	50.5±14.4	55.4±13.4^★^	57.8±12.4^★▲^	59.2±11.7^★▲^	<0.001
History of hypertension (%)	87 (16.8)	151 (29.1)	168 (32.2)	209 (40.4)	<0.001
History of diabetes (%)	24 (4.6)	41 (7.9)	43 (8.3)	49 (9.5)	0.02
History of CHD (%)	13 (2.9)	15 (2.5)	26 (5.0)	23 (4.4)	0.10
History of stroke (%)	4 (0.8)	10 (1.9)	6 (1.2)	10 (1.9)	0.30
Education of high school or above (%)	215 (45.3)	170 (35.6)	153 (32.1)	164 (34.8)	<0.001
Physical inactivity (%)	205 (39.6)	207 (39.9)	168 (32.2)	179 (34.6)	0.02
Current smoking (%)	37 (7.5)	56 (11.2)	69 (13.7)	87 (17.5)	<0.001
Current alcohol consumption (%)	15 (3.1)	9 (1.8)	33 (6.6)	39 (7.9)	<0.001
RBP4 (mg/L)	39.5±4.8	50.3±2.3^★^	58.9±2.9^★▲^	76.1±11.8^★▲◆^	<0.001
SBP (mmHg)	125.1±18.8	134.9±19.7^★^	135.5±18.8^★^	141.1±18.6^★^	<0.001
DBP (mmHg)	78.1±10.0	82.6±10.2^★^	83.2±10.3^★^	86.1±10.6^★▲◆^	<0.001
WC (cm)	79.1±9.6	83.8±9.2^★^	86.6±9.6^★▲^	89.1±8.6^★▲◆^	<0.001
BMI (kg/m^2^)	22.8±3.2	23.9 ± 3.3^★^	24.7±3.5^★▲^	25.4±3.2^★▲◆^	<0.001
TG (mmol/L)	0.93 (0.75–1.19)	1.19 (0.94–1.50) ^★^	1.37 (1.07–1.81) ^★▲^	2.25 (1.65–2.98) ^★▲◆^	<0.001
LDL (mmol/L)	2.91±0.79	3.27±0.86	3.42±0.93^★▲^	3.31±1.08^★▲◆^	<0.001
HDL (mmol/L)	1.60±0.33	1.54±0.32^★^	1.53±0.35^★^	1.36±0.34^★▲^	<0.001
eGFR (mL/min/1.73 m^2^)	92.8±15.1	87.1±15.7^★^	2.1±15.2^★▲^	77.6±17.2^★▲◆^	<0.001
hs-CRP (mg/L)	0.96 (0.00–1.84)	1.35 (0.60–2.49) ^★^	1.54 (0.70–2.82)^★^	1.73 (0.92–2.93)^★▲◆^	<0.001
FBG (mmol/L)	4.95±0.92	5.21±1.17^★^	5.34±1.16^★^	5.54±1.49^★◆^	<0.001
Insulin (μU/mL)	7.35 (5.61–10.60)	8.81 (6.55–12.34) ^★^	9.39 (6.64–13.40) ^★▲^	11.62 8.36–16.06) ^★▲◆^	<0.001
HOMA-IR (µU/mL ·mmol/mL)	1.61 (1.17–2.30)	1.97 (1.46–2.92) ^★^	2.15 (1.46–3.32) ^★▲^	2.70 (1.90–3.99) ^★▲◆^	<0.001
Serum uric acid (µmol/L)	290.1±63.7	337.3±77.3^★^	364.8±82.0^★▲^	405.9 ± 93.4^★▲◆^	<0.001
ACR (mg/g)	10.4 (7.0–16.5)	10.0 (6.7–18.5)	10.3 (6.8–18.4)	12.7 (6.9–28.0)^★▲◆^	0.01
Cystatin C (mg/L)	0.84±0.16	0.90±0.18^★^	0.95±0.21^★▲^	1.02±0.29^★▲◆^	<0.001
NAG (U/L)	2.50 (1.30–4.30)	2.80 (1.60–4.72) ^★^	2.90 (1.50–5.50) ^★^	3.20 (1.90–5.70) ^★▲^	<0.001
IL-6 (pg/mL)	2.93 (2.40–4.00)	3.30 (2.48–4.50) ^★^	3.38 (2.67–4.49) ^★^	3.37 (2.76–4.48) ^★^	<0.001
β2MG (µg/mL)	0.08 (0.05–0.14)	0.10 (0.05–0.17)	0.09 (0.05–0.17)	0.10 (0.05–0.17)	0.23

Data are shown as mean±standard deviation, median (interquartile range), or frequency (percentage).

★ vs. quartile 1, P < 0.05; ▲ vs. quartile 2, P < 0.05; ◆ vs. quartile 3, P < 0.05.

The prevalence of hyperuricemia gradually increased from the first to the fourth RBP4 quartile from 5.0% to 58.2% (7.5% to 61.7% in males and 4.4% to 55% in females) (all *P*<0.001, **Table 3**).

**Table 3:** Prevalence of hyperuricemia by RBP4 quartile .

Characteristic	Quartile 1	Quartile 2	Quartile 3	Quartile 4	χ^2^	*P*
Total (*n =*2075)	*n*= 518	n = 519	n = 521	n = 517		
Hyperuricemia (%)	26 (5.0)	128 (24.7)	196 (37.6)	301 (58.2)	360.5	<0.001
Non-hyperuricemia (%)	492 (95.0)	391 (75.3)	325 (62.4)	216 (41.8)		
Male (*n =*744)	106	172	218	248		
Hyperuricemia (%)	8 (7.5)	49 (28.5)	87 (39.9)	153 (61.7)	104.7	<0.001
Non-hyperuricemia (%)	98 (92.5)	123 (71.5)	131 (60.1)	95 (38.3)		
Female (*n =*1331)	412	347	303	269		
Hyperuricemia (%)	18 (4.4)	79 (22.8)	109 (36.0)	148 (55.0)	231.8	<0.001
Non-hyperuricemia (%)	394 (95.6)	268 (77.2)	194 (64.0)	121 (45.0)		

The associations between RBP4 and hyperuricemia, according to six multivariate binary logistic regression analyses, are shown in **Table 4**. RBP4 and hyperuricemia were positively associated in all six models. In the final model (model 6), the OR comparing quartile 4 of RBP4 with quartile 1 was 7.90 (95% CI: 4.18–14.84; *P* < 0.001).

**Table 4:** Association between RBP4 and hyperuricemia according to logistic regression.

Characteristic	Quartile 1	Quartile 2	Quartile 3	Quartile 4	*P* (trend)
	*n*= 518	*n*= 519	*n*= 521	*n*= 517	
	Reference	OR (95% CI)	OR (95% CI)	OR (95% CI)	
Model 1	1.00	5.8 (3.73–9.07)	10.3 (6.66–15.97)	23.3 (15.04–36.12)	<0.001
Model 2	1.00	5.3 (3.32–8.34)	9.7 (6.20–15.29)	20.8 (13.24–32.80)	<0.001
Model 3	1.00	4.5 (2.75–7.22)	7.0 (4.33–11.26)	11.6 (7.13–18.78)	<0.001
Model 4	1.00	4.5 (2.75–7.20)	6.8 (4.22–10.94)	11.3 (6.94–18.24)	<0.001
Model 5	1.00	4.3 (2.44–7.45)	6.2 (3.52–10.79)	8.1 (4.38–14.88)	<0.001
Model 6	1.00	4.6 (2.59–8.17)	6.0 (3.37–10.67)	7.9 (4.18–14.84)	<0.001

Regression model 1: adjusted for age and sex.

Regression model 2: adjusted for model 1 covariates plus medical history (hypertension, diabetes, and CHD), education of high school or above, physical inactivity, current smoking, and current alcohol consumption.

Regression model 3: adjusted for model 2 covariates plus SBP, DBP, log TG, LDL, HDL, BMI, eGFR, FBG, and log insulin.

Regression model 4: adjusted for model 2 covariates plus SBP, DBP, log TG, LDL, HDL, BMI, eGFR, and log HOMA-IR.

Regression model 5: adjusted for model 4 covariates plus log hs-CRP, and log IL-6.

Regression model 6: adjusted for model 5 covariates plus log NAG and log ACR.

**Figure 1:**
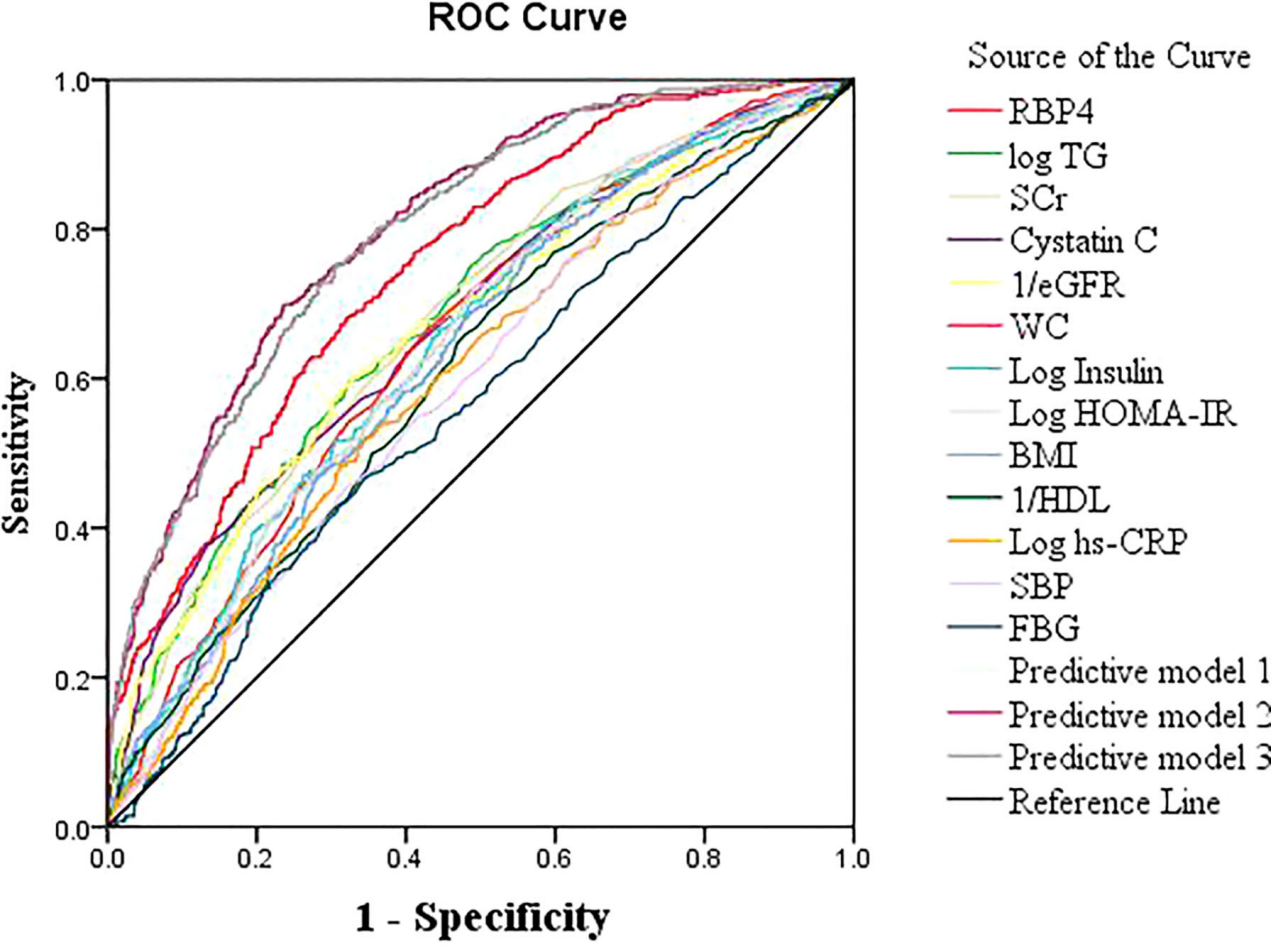
Receiver operating characteristic (ROC) curves of RBP4 and other indicators for predicting hyperuricemia in the general population.

The AUC for RBP4 predicting hyperuricemia was 0.749 (95% CI: 0.725–0.774, *P*<0.001) and Youden’s index was 0.36, with an optimum cutoff of 54.5 mg/L. Its predictive performance was better than the performances of SCr, cystatin C, eGFR, TG, WC, BMI, insulin, HOMA-IR, HDL, FBG, hs-CRP, and SBP (**Figure 1**).

The best prediction model was model 3, which involved RBP4 and other variables related to uric acid (sex, BMI, SCr, hs-CRP, FBG, insulin, and current alcohol consumption) in a binary logistic regression model (AUC: 0.804, 95% CI: 0.782–0.826, Youden’s index: 0.36, *P*<0.001). The predictive power of model 1 (AUC: 0.803) and model 2 (AUC: 0.797) were close to that of model 3 (**Table 5**; **Figure 1**). The best predictive model for males was composed of RBP4, SCr, and BMI (AUC: 0.782, 95% CI: 0.749–0.815, Youden’s index: 0.424, *P*<0.001). The best predictive model for females was composed of RBP4, SCr, hypertension, log insulin, and log hs-CRP (AUC: 0.824, 95% CI: 0.796–0.852, Youden’s index: 0.510, *P*<0.001).

**Table 5:** Predictive value of RBP4 and other indicators for hyperuricemia in the general population.

Characteristic	AUC	95% CI	*P* value	Sensitivity	1-Specificity	Youden’s index	Cutoff
RBP4	0.749	0.725–0.774	<0.001	0.681	0.321	0.360	54.5
Log TG	0.682	0.654–0.710	<0.001	0.596	0.325	0.271	0.18
Serum creatinine	0.680	0.652–0.707	<0.001	0.697	0.440	0.257	72.5
Cystatin C	0.677	0.649–0.704	<0.001	0.453	0.208	0.245	1.00
WC	0.653	0.625–0.681	<0.001	0.665	0.429	0.236	85.8
1/eGFR	0.668	0.640–0.697	<0.001	0.591	0.319	0.272	0.012
Log insulin	0.646	0.618–0.674	<0.001	0.806	0.585	0.221	0.89
BMI	0.634	0.606–0.663	<0.001	0.766	0.562	0.205	23.4
Log HOMA-IR	0.644	0.616–0.672	<0.001	0.719	0.489	0.230	0.30
FBG	0.564	0.535–0.594	<0.001	0.467	0.346	0.122	5.21
Log hs-CRP	0.601	0.572–0.631	<0.001	0.516	0.342	0.175	0.32
1/HDL	0.610	0.581–0.639	<0.001	0.659	0.479	0.180	0.67
SBP	0.596	0.567–0.625	<0.001	0.757	0.617	0.141	126.5
Predictive model 1	0.803	0.781–0.825	<0.001	0.701	0.239	0.462	0.36
Predictive model 2	0.797	0.775–0.819	<0.001	.754	.308	0.446	0.31
Predictive model 3	0.804	0.782–0.826	<0.001	.699	.237	0.462	0.36

Predictive model 1: RBP4, sex, BMI, SCr, log hs-CRP, log insulin, log HOMA-IR, and current alcohol consumption.

Predictive model 2: RBP4, sex, BMI, SCr, log hs-CRP, and FBG.

Predictive model 3: RBP4, sex, BMI, SCr, log hs-CRP, FBG, log insulin, and current alcohol consumption.

## Discussion

Using population-based data on southern Chinese adults collected in a single-center cross-sectional epidemiological survey, we found that RBP4 had a highly significant association with hyperuricemia (*P*<0.001). More importantly, RBP4 was a good predictor of hyperuricemia; indeed, it performed better than traditional predictors. We also explored the predictive value of RBP4 combined with routinely assessed clinical factors that are related to hyperuricemia. To our knowledge, this is the first study to investigate the predictive value of RBP4 for hyperuricemia.

Several groups have reported individual predictors or predictive models for hyperuricemia (20–22). Lee MF et al. (20) reported that sex, BMI, and peroxisome proliferator-activated receptor (PPAR)-γ polymorphism are good predictors of hyperuricemia. Cao et al. (21) developed sex-specific prediction models (the predictors for males were age, SBP, BMI, and blood uric acid, and the predictors for females were SBP, BMI, TG, and blood uric acid) for hyperuricemia. Lee S et al. (22) developed a machine learning model (involving basic healthcare checkup test results) for predicting hyperuricemia. In addition, various novel blood lipid indicators such as the visceral adiposity index (18, 33), TG-glucose index (19), lipid accumulation products (34), and the cardiometabolic index (35) have been independently associated with hyperuricemia. However, all these studies were limited to specific populations, and the results were not verified in other regions such as the southern China. For example, Lee MF et al. (20) focused on adults aged 20–40 years in a district of Taiwan, China; the machine learning model (22) was based on a Korean population; and the prediction model developed by Cao et al. (21) was based on urban Han Chinese adults in Shandong province. Additionally, the predictive values of most of the above individual predictors (e.g., TG-glucose index, AUC: 0.662) or models (e.g., Cao et al. (21), AUC: 0.783) were relatively poor. Moreover, some of the predictors (e.g., PPAR-γ polymorphism) are not suitable for clinical use or for use in large-scale epidemiological research due to their high cost and complexity.

RBP4, as a single factor, had a good performance in predicting the risk of hyperuricemia. The AUC for RBP4 in the general population was 0.749. This is better than previous traditional individual predictors and similar to the predictive model developed by Lee MF et al. (20), which had an AUC of 0.775. We also obtained a predictive model (model 3), involving RBP4 and traditional predictors, with a good AUC of 0.804. Notably, the predictive value of RBP4 was slightly better in females than males (AUC for RBP4: 0.738 vs. 0.756; AUC for predictive model 3: 0.782 vs. 0.824).

The conclusion of our investigation is consistent with two previous studies (24, 25). Chen et al. were the first to report elevated serum RBP4 levels with increasing serum uric acid among 885 individuals with type 2 diabetes in Taiwan. Thereafter, Chan et al. (25) reported that serum RBP4 was positively associated with uric acid in 26 subjects with hypertension and MetS.

Our study has the following strengths. First, the participants were randomly sampled from the general population and covered all ages of adults, so the sample is widely representative. Second, 2075 people were included in the analysis, far exceeding the sample sizes of previous studies. Third, strict eligibility criteria were adopted. For example, we excluded individuals who were treated with uric acid-lowering drugs and those with a history of diseases or medications that might affect the RBP4 level. Fourth, the association remained significant after adjustment. For example, after adjusting for 21 potential confounders, the final regression model still showed that the risk of hyperuricemia in the highest RBP4 quartile was still 7.9 times higher than that in the lowest quartile, indicating a significant independent association between RBP4 and hyperuricemia. Finally, this is the first study to evaluate the value of RBP4 for predicting the risk of hyperuricemia, and we found that RBP4 alone performed better than traditional predictors. This is also the most important finding of this study, as it indicates that RBP4 might be useful for predicting the risk of hyperuricemia and could be used in epidemiological research on hyperuricemia in general adult populations. Though direct assessment of uric acid should be prefered over measuring a surrogate, the association between RBP4 and uric acid could be diagnostically exploited in case uric acid values are not available.

The mechanisms by which how RBP4 predicts hyperuricemia remain unclear. RBP4 is considered independently related to insulin resistance, which is implicated in the pathogenesis of hyperuricemia (36, 37). A recent study showed that RBP4 might be involved in hyperuricemia-induced insulin resistance by inhibiting IRS/PI3K/Akt phosphorylation (38).

Even after adjusting for insulin level plus FBG, or HOMA-IR, RBP4 remained strongly associated with hyperuricemia. This indicates that RBP4 may be related to hyperuricemia through other mechanisms besides insulin resistance. This study also indicated that the mechanisms may not involve renal function. When we adjusted for all renal function indicators (including glomerular and renal tubular function; i.e., eGFR, ACR, and NAG) in regression model 6, the regression results showed that the independent association between RBP4 and hyperuricemia remained significant. We speculate that besides insulin resistance, RBP4 may have additional unique non-renal function mechanisms such as pro-inflammatory effects and direct effects on vascular smooth muscle and uric acid (23, 39, 40). Further basic research on the mechanisms is needed in the future.

Our results should be considered in the context of several limitations. First, the participants were all Han Chinese adults from Zhuhai city, and the results may not be generalizable to other ethnicities. In addition, this was a single-center study and therefore inevitably limited regarding the sample size; large multicenter studies are needed to verify the conclusions. Furthermore, the study was cross-sectional, and the underlying mechanisms were not explored in depth. Cohort or case-control studies and basic research on the underlying mechanisms should be performed to verify our findings.

## Conclusions

This study found that RBP4 is significantly positively associated with hyperuricemia in adults and has good predictive value for the condition. Clinically, it can be used alone or in combination with other traditional indicators to predict the risk of hyperuricemia.

## Data Availability Statement

The raw data supporting the conclusions of this article will be made available by the authors, without undue reservation.

## Ethics Statement

The studies involving human participants were reviewed and approved by Ethics Committee of the Third Affiliated Hospital of Southern Medical University. The patients/participants provided their written informed consent to participate in this study. Written informed consent was obtained from the individual(s) for the publication of any potentially identifiable images or data included in this article.

## Author Contributions

G-BH, HZ, and C-XX participated in the research design, performance of the research, data analysis, and manuscript writing. X-FS, QZ, and X-SK participated in the research design and data analysis. P-CG, X-LL, JN, H-SC, and HX participated in the performance of the research and data analysis. All authors were involved in revising the manuscript and approved the final version.

## Funding

This study was supported by the following sources, (1) Clinical Research Startup Program of Southern Medical University by High-level University Construction Funding of Guangdong Provincial Department of Education (LC2016PY047, 2016, ChiCTR1800016248), (2) Self-financing Science and Technology Projects of Foshan City (Medical Science and Technology Research; 1920001001357, 2019), (3) Science and Technique Program of Guangzhou (201604020015, 2015), (4) South Wisdom Valley Innovative Research Team Program (CXTD-004, 2014), (5) Guangdong Provincial Science and Technique Program (2011B031800386, 2011), (6) ISN Research Committee grant (2007), and (7) EU FP7 Program (UroSense, 2011).

## Conflict of Interest

Author P-CG was employed by Nanjing CR Medicon Pharmaceutical Technology Co., Ltd.

The remaining authors declare that the research was conducted in the absence of any commercial or financial relationships that could be construed as a potential conflict of interest.

## Publisher’s Note

All claims expressed in this article are solely those of the authors and do not necessarily represent those of their affiliated organizations, or those of the publisher, the editors and the reviewers. Any product that may be evaluated in this article, or claim that may be made by its manufacturer, is not guaranteed or endorsed by the publisher.

## Abbreviations

ACR, albumin-to-creatinine ratio; β2MG, β2 microglobulin; BMI, body mass index; CHD, coronary heart disease; DBP, diastolic blood pressure; eGFR, estimated glomerular filtration rate; FBG, fasting blood glucose; HDL, high-density lipoprotein; HOMA-IR, homeostasis model assessment of insulin resistance; hs-CRP, high sensitivity C-reactive protein; IL-6, interleukin 6; LDL, low-density lipoprotein; NAG, N-acetyl-β-D-glucosaminidase; RBP4, retinol binding protein 4; SBP, systolic blood pressure; SCr, serum creatinine; TG, triglycerides; WC, waist circumference.
